# Pervasive assistive technology for people with dementia: a UCD case

**DOI:** 10.1049/htl.2016.0057

**Published:** 2016-11-02

**Authors:** Julia Rosemary Thorpe, Kristoffer V.H. Rønn-Andersen, Paulina Bień, Ali Gürcan Özkil, Birgitte Hysse Forchhammer, Anja M. Maier

**Affiliations:** 1 Department of Management Engineering, Technical University of Denmark, Kgs. Lyngby 2800, Denmark; 2 Department of Mechanical Engineering, Technical University of Denmark, Kgs. Lyngby 2800, Denmark; 3 Department of Neurology, Rigshospitalet-Glostrup, Glostrup 2600, Denmark

**Keywords:** smart phones, diseases, application usage logs, logbooks, system usability scale questionnaires, interaction logs, video recordings, field testing, smartwatch, off-the-shelf pervasive technologies, user-centred design methods, people-with-dementia, wearable technology, smart mobile, UCD case, pervasive assistive technology

## Abstract

Smart mobile and wearable technology offers exciting opportunities to support people with dementia (PwD). Its ubiquity and popularity could even benefit user adoption – a great challenge for assistive technology (AT) for PwD that calls for user-centred design (UCD) methods. This study describes a user-centred approach to developing and testing AT based on off-the-shelf pervasive technologies. A prototype is created by combining a smartphone, smartwatch and various applications to offer six support features. This is tested among five end-users (PwD) and their caregivers. Controlled usability testing was followed by field testing in a real-world context. Data is gathered from video recordings, interaction logs, system usability scale questionnaires, logbooks, application usage logs and interviews structured on the unified theory of acceptance and use of technology model. The data is analysed to evaluate usability, usefulness and user acceptance. Results show some promise for user adoption, but highlight challenges to be overcome, emphasising personalisation and familiarity as key considerations. The complete findings regarding usability issues, usefulness of support features and four identified adoption profiles are used to provide a set of recommendations for practitioners and further research. These contribute toward UCD practices for improved smart, pervasive AT for dementia.

## 1 Introduction

An ageing population spells a rise in the prevalence of dementia which will greatly impact quality of life for the elderly [1]. While assistive technology (AT) could potentially support people with dementia (PwD), it is highly fragmented with very low adoption in practice. An interesting solution may lie at arm's reach: readily available smartphones and wearables could be used to merge many of the roles of existing AT on a flexible and popular platform.

Mainstream pervasive technologies have successfully been applied in healthcare applications within the growing field of pervasive healthcare [2]. While opportunities for pervasive healthcare in dementia are extensive [3], little is known about whether PwD are interested in or able to use smart products since these predominantly target the young and healthy. For people in the early stages of dementia who live at home, these technologies are particularly interesting for keeping users independent and socially engaged.

Several works evaluate usability of AT for cognitively impaired users; however, these tend to focus on specialised equipment developed for the impaired [4]. The aim of this work is therefore to investigate adoption among users with mild dementia of a pervasive AT solution using only off-the-shelf technology. We first develop a basic solution by selecting and combining a smartwatch, smartphone and applications (apps). This is tested among users to evaluate usability, usefulness and acceptance, highlighting key issues regarding smart mobile and wearable devices for PwD. The results are used to draw up recommendations for design and healthcare practitioners, identify adoption profiles and highlight important areas for future research.

### 1.1 User-centred design (UCD) approach

UCD aligns neatly with patient-centred care and is essential in developing successful AT for dementia, though it has often been overlooked [5]. Inclusive design and co-design are two related concepts that tie into UCD. Inclusive design has contributed substantially toward designing for elderly users over the years [6]. Research on co-design methods for users with cognitive impairment encourages involvement of end-users and the use of simple prototypes [7]. Several studies developing AT for dementia document a UCD approach that involves users and includes evaluation of usability and usefulness [8–12]. Though important, these examples apply UCD methods to develop specialised AT equipment rather than employ off-the-shelf solutions. In this work, we will instead focus on adapting consumer-grade pervasive technology. To do so, we employ a similar UCD approach that incorporates user testing guided by inclusive design [13, 14] and factors influencing technology adoption [15].

The people, activities, context and technology (PACT) framework [16] can guide the evaluation of a system involving human–technology interaction. Accordingly, user testing should involve the target group (*people*) using the *technology* for its intended purpose (*activities*) in realistic *contexts*. This can be achieved by combining different types of user testing in both a controlled environment and real-world context such as usability testing and field testing [13].

The system usability scale (SUS) is a standardised questionnaire used to evaluate the perceived usability of the solution among the users [17, 18]. In addition to usability, perceived usefulness is also important for technology adoption among elders [19]. These relate to *complexity* and *relative advantage*, which are two characteristics of innovations that influence their adoption [20]. The unified theory of acceptance and use of technology (UTAUT) is an established model for evaluating technology adoption [21]. This includes questions on usefulness and usability, as well as on social influence, facilitating conditions, intention to use and use behaviour. These align with findings from a study on hearing aids (another AT that targets older users) in which adoption barriers included low perceived severity, stigmatisation, technology anxiety and low usability due to poor vision or dexterity [22]. The use of popular, pervasive technology could influence these factors when it comes to AT for dementia, and is described in the following section.

### 1.2 Pervasive AT for dementia

AT for dementia covers a wide range of technological concepts including smart homes, tracking devices and interactive systems. These are used to provide functional support with memory and everyday activities, for social engagement or stimulation and to improve safety and care. The use of pervasive computing for AT is referred to as pervasive AT [2]. In this work, the focus is on smart mobile and wearable devices, specifically smartphones and smartwatches, which can serve as both interactive and tracking devices. These products are inherently flexible due to their modular nature: a device can employ different combinations of sensors and apps to serve a particular function or set of functions. The popularity, ubiquity and flexibility of pervasive products could enhance adoption among PwD. Compared with other specialised aids; we recognise the following key advantages offered by products such as smartphones and wearables:

- They are *less stigmatised* since they do not draw attention to the user's impairment.
- Increasingly in future, they will be *familiar* to users who already rely on these technologies in their everyday lives prior to their cognitive impairment, making them easier to learn to use.
- They are well known and *available* worldwide, making them more accessible than products that users may not be able to purchase in their home country – or may never hear of.
- Their flexibility lends itself to personalisation. Products can be selected and customised to *fit individual needs* and adapted as these change over time at minimal cost or effort (e.g. by installing a new app or adjusting settings).
- They are more ‘*future proof*’. Changes or upgrades to the user's smartphone, wearable or apps as technology improves should not hinder the provision of continued functional support, except where version compatibility is affected.

AT should also meet users’ support needs to be useful. Indeed, smart technology and wearables could meet many of the needs of PwD, their caregivers and healthcare providers. In earlier work, four main need areas have been identified as: functional, psychosocial, safety and care needs [23]. These were identified from the literature as well as through needs gathering activities (interviews, observation and shadowing) involving multiple stakeholders (PwD, their caregivers and healthcare professionals). Many of the support features offered by existing AT to meet these need areas are readily available from common pervasive technology. A selection of six key support features includes:

- *Scheduling*: Calendars that provide an overview of a user's schedule and reminders for tasks and appointments [12, 24].
- *Navigation*: Assist a user in finding their way [25, 26]. This could also improve mobility and keep users socially engaged.
- *Communication*: Enabling users to easily contact their primary caregiver and others in their care and social networks [26–28].
- *Orientation*: Provide current time of day and location (temporal and spatial orientation), since a PwD can become disoriented [29].
- *Emergency help*: Provide a way for users to elicit help in the case of an emergency such as a fall or when they are lost [26, 30].
- *Monitoring*: Gather behavioural data to measure indicators of the user's status and/or wellbeing [31, 32]. This can support healthcare professionals in delivering timely and appropriate care.

There are many other roles of AT for dementia that could be fulfilled using pervasive technology, e.g. fall detection, activity recognition, life-logging and reminiscence therapy to name a few [5, 33, 34]. The above support features are selected as a starting point based on both being relatively simple to implement with available apps and offering considerable benefit for users.

## 2 Equipment selection and adaptation

The equipment selected to fulfil the support features outlined in the previous section includes a smartphone and smartwatch used in combination with various apps. The smartphone provides a familiar interface through which users can input data and carry out tasks. The smartwatch provides a second interface and wearable sensors located on the wrist. This is especially relevant for PwD who may have difficulty remembering, since the user need not remember to carry their smartphone on them at all times in their home. Inclusive design and personalisation were focal points in the selection of devices and influenced the choice of operating system (OS) and physical hardware.

Personalised support that fits the user and their individual needs is important for user adoption. In choosing an OS, this meant considering device compatibility, support for a wide range of apps and adaptability; on the basis of which the Android platform was selected. All smartwatches built with the Android Wear platform are compatible with Android 4.3+ or iOS 8.2+, offering a wider selection of watch designs than Apple's iOS. Android (and Android Wear) also offer a wide selection of apps and greater opportunity for adaptation and customisations.

**Fig. 1:**
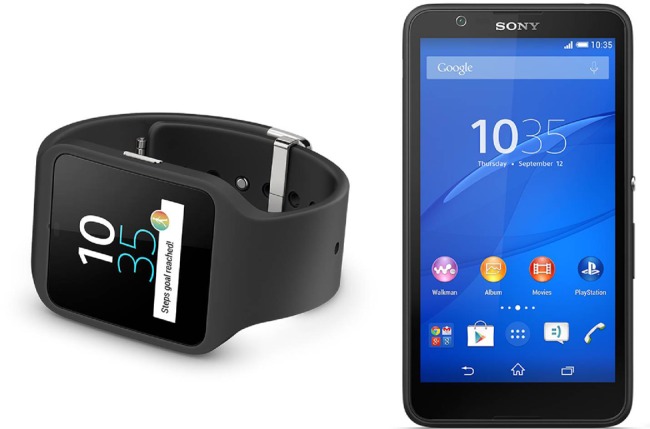
Selected hardware: Sony SmartWatch 3 (left) and Sony Xperia E4 (right)

Inclusive design guided the hardware selection. Primary end-users include the PwD and their caregivers (usually a spouse or child) indicating an elderly user group, since most PwD are over 65 years old. Capabilities of elderly users such as their vision, hearing and dexterity relate to usability principles such as visibility, affordance and feedback [13]. This led to the following priorities: a large screen for visibility; touch input rather than buttons for improved affordance; tactile feedback; and the ability to provide haptic (vibration) notifications. Battery life exceeding 1 day was also considered important such that the user can routinely charge the device at night. This takes into account the PwD memory impairment, since structured routines make tasks easier to remember. On the basis of these considerations, the Sony Smartwatch 3 and Sony Xperia E4 devices were selected and are shown in Fig. 1. The smartwatch runs Android Wear and the smartwatch Android OS v4.4.4.

Following hardware selection, the devices were adapted to meet each of the six support features described in Section 1.2. This involved installing selected off-the-shelf apps and widgets, and customising the phone and watch home screens. An overview of the software setup used to provide each support feature is outlined in Table 1. Of these, *monitoring* was not tested further, since this is not used directly by the PwD or caregiver. It is included up to this point to indicate how the pervasive AT solution could be applied to gather data for further measurement, since this is of great interest among AT for dementia.

**Table 1:** Overview of software setup on the phone and watch to achieve six support features

Function	Application	Device	Purpose
scheduling	Google Keep	P	Create notes and lists with reminders, displayed on phone screen using a widget
	Google Calendar	P	Manage schedule by entering appointments and events on the phone or web portal
	DigiCal Widget	P	Display schedule and notify about events on the smartphone screen
	Agenda	W	Calendar overview on smartwatch, synced with Google Calendar
navigation	Google Maps	P, W	Route planning and turn-by-turn navigation
communication	contact widget	P	Shortcut to call primary caregiver displayed on the home screen
orientation	AccuWeather	P	Display time and location on home screen
	Custom watchface	W	Display time of day (e.g. ‘Thursday afternoon’) together with time on watchface
emergency help	IFTTT	W, P	Shortcut for user to send caregiver an emergency message with their location
			notify caregiver when the user leaves a predefined safe-zone
monitoring	moves	P	Track user's mobility (out of home)
	fit	P, W	Track user's activity levels (in and out of home)

Abbreviations: Phone (P), Watch (W).

## 3 User testing

The prototype was tested with target users both in a controlled setting and a real-world context. Participants were recruited through a dementia clinic at a Danish hospital and participated together with their spouses as pairs. The only criterion for inclusion was a diagnosis of mild cognitive impairment or mild dementia since this is the target group for the pervasive AT which, should be introduced as early as possible in the disease process. Participation was voluntary and with informed consent. In total, six pairs were recruited and will be referred to using the codes P1–P6 (for PwD numbered 1–6). P4 dropped out at the start and the remaining five pairs included three male and two female PwD between the ages of 61 and 73 years, four of whom claim to use a smartphone daily (two Android, two iOS) and one with no experience.

**Fig. 2:**
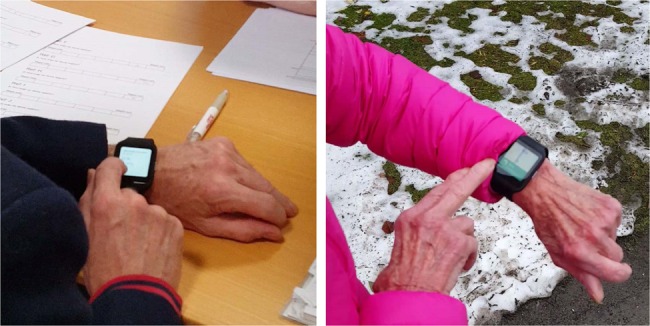
Participants performing tasks on the smartwatch during the usability testing

The user testing was implemented in two phases: usability testing and field testing. The goal of the usability testing is to identify key usability issues and their causes. This was facilitated by a project member and followed a predefined protocol involving 13 tasks over ∼2 h in a controlled environment (see Fig. 2). Interaction logs and video recordings were used to collect data on task completion rates, and on the frequency and severity of errors or problems. An SUS questionnaire was used to assess perceived usability at the end of each phase.

The field testing allowed testing ‘in the wild’. In this phase, the participant pairs took the devices home and used them unsupervised for 1 week. The goal of field testing was to assess perceived usability and usability issues in real life, and to assess acceptance and use, and any issues that influence these. Several methods were used to collect data: participants kept logbooks to note down issues and experiences, application usage was logged on the devices, and a semi-structured debriefing interview was performed. The debriefing interview was guided by the UTAUT model and also included a repeat of the SUS questionnaire.

## 4 Analysis and results

**Fig. 3:**
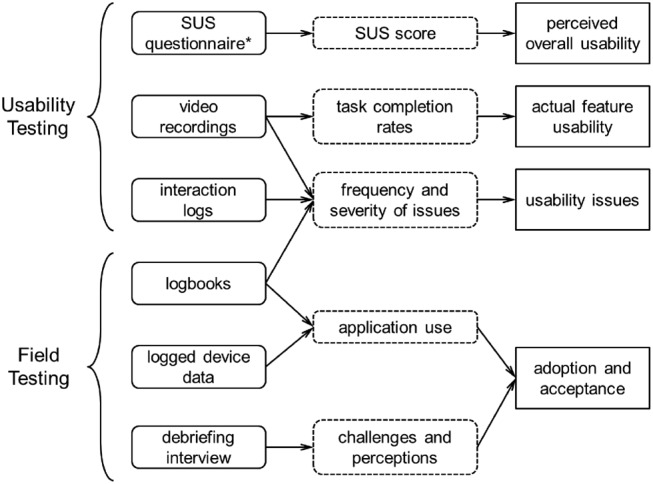
Overview of the data collection methods for each part of the testing. *SUS was repeated during the debriefing interview following field testing

Data collected in the user testing was analysed to evaluate: perceived overall usability, actual usability by support feature, key usability issues and user acceptance. Fig. 3 provides an overview of the various data sources and how these contribute to these analyses.

### 4.1 Perceived overall usability

**Fig. 4:**
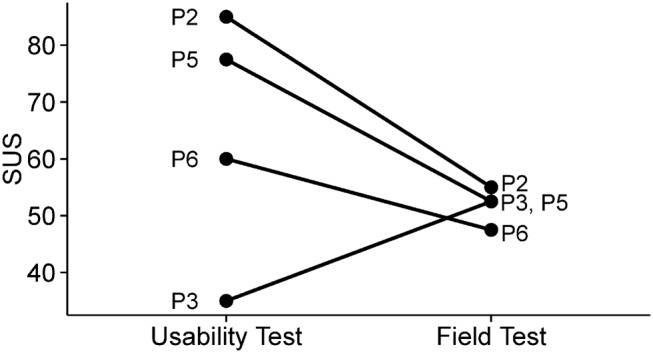
System usability scale (SUS) scores for four participants, measured after the usability testing and then after the field testing

The SUS score measured the user's perception of the usability of the system as a whole following both the usability and field tests. P1 did not attend the debriefing; therefore, both scores are only available for four participants. The results are shown in Fig. 4, which indicates that agreement among participants regarding perceived usability was higher after the field testing. SUS scores decreased following the field testing for three participants, with only P3's improving (however, this was extremely low at the usability test). Interestingly, P3 was also the only participant without prior experience using a smartphone.

### 4.2 Actual usability by feature

Video recordings of the usability testing were analysed to calculate completion rates for each of the tasks performed by the participants. The tasks relate to the features described in Section 1.2 (scheduling, navigation, communication, orientation and emergency help), as well as to more general use of the smartphone and smartwatch. This complements the SUS scores by providing objective information on usability and detailing this for specific aspects of the prototype rather than the overall system. The task completion rates were organised into three tiers according to whether they were completed by all participants, some participants or no participants. An overview of the results is provided in Table 2, indicating which support feature relates to the tasks listed.

**Table 2:** Completion rates for tasks performed by users during the usability testing, given as the number of participants who successfully completed the tasks out of a total of five participants

Feature	Tasks	Completion rate
scheduling	use calendar	5/5
	notifications	
communication	call partner	
orientation	orientation	
general	unlock screen	
	equip watch	

general	charge watch	4/5
scheduling	see agenda	2/5
	use to-do list	

emergency help	use emergency contact	0/5
navigation	navigation	

### 4.3 Key usability issues

Data on problems or errors was collected from the facilitator's interaction logs (usability testing) and participants’ logbooks (field testing). This was analysed according to both frequency and severity of usability issues to identify the most critical issues. These are described below, grouped according to the task or component to which they relate.

- Smartphone and smartwatch interfaces:
  - *Swiping:* User does not intuitively swipe to see widgets on the smartphone or know when to stop swiping through the menu on the smartwatch.
  - *Home screens/watchfaces:* Users accidentally edit or delete home screens (phone) and watchfaces (watch) by pressing them for too long.
- *Smartwatch charging:* Users had difficulty locating the charging port and inserting the cable.
- *Navigation:* Users need to start moving in order to calculate their direction, causing them to start off in the wrong direction.
- *Smartphone calling:* Users familiar with smartphones used the contact book rather than the shortcut to call their caregiver.
- Reminders and notifications:
  - Reminders require different notice periods (e.g. a doctor appointment requires time to prepare and travel, medication reminders do not), which was confusing for users to handle.
  - The use of multiple apps (Google Calendar, Google Keep, DigiCal and Agenda) for *scheduling* caused confusion.
  - Several participants recorded blank notes in Google Keep, possibly due to voice enabled note-taking on the smartwatch.
- *Device pairing:* The Bluetooth connection between the phone and watch drops unexpectedly; or is completely lost, requiring a factory reset on the smartwatch to be re-established.
- *Unintuitive application names/icons:* Users had difficulty finding the functionality they were looking for based on the icon/name, e.g. where the application If This Then That (IFTTT) was used to create an emergency help feature.

### 4.4 User adoption and acceptance

**Fig. 5:**
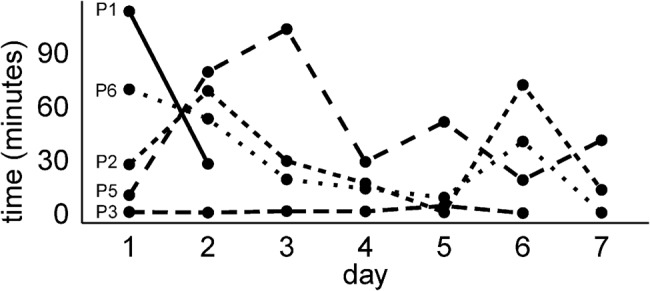
Time participants spent using the AT solution during field testing based on logged app use

The analysis of user adoption and acceptance is based on data collected in the field testing only. Data on app usage was logged on the devices throughout the field testing as an indication of how much participants actually used the solution, which is depicted in Fig. 5. During the debriefing interview, participants were asked whether they found the prototype useful and easy to use, if they felt encouraged to use it by their social/care network, were adequately supported in using it, and about their intention to use the prototype further (as per the UTAUT model).

**Fig. 6:**
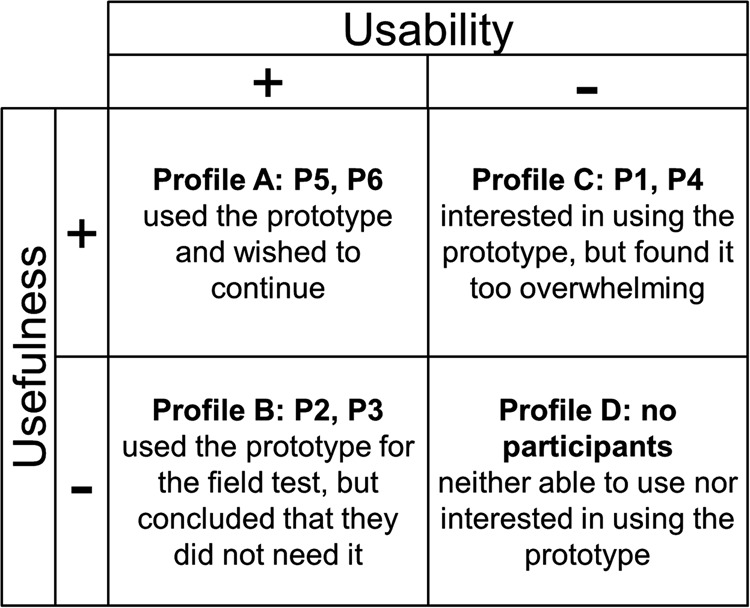
Adoption profiles based on data collected during the debriefing interviews

Information from the logged data, debriefing interview and general participant feedback was used to place the participants within four identified adoption profiles summarised in Fig. 6. For P1 and P4 (Profile C), usability was a key adoption barrier that even prevented them from participating for the duration of the study. P2 and P3 (Profile B) were able to use the prototype, but felt they did not need it or preferred their current strategy (e.g. using a wall calendar). Here, low utility or usefulness is the key barrier. More specifically, the solution does not replicate familiar or established methods. Profile A includes P5 and P6 who accepted the solution and wished to use it further. These profiles refer to two broad adoption barriers: usability and usefulness. Usability is dealt with in detail through the previous analyses. Regarding usefulness, several key points were noted from participant feedback:

- None of the participants felt that they needed the navigation support.
- Reminders were considered highly useful by both participants who wished to continue using the prototype (Profile A).
- Interoperability was a considerable barrier for the iPhone user.

These collected findings and their implications for practitioners and future research efforts are discussed in the following section.

## 5 Discussion

Our findings have uncovered valuable insights regarding both the methods and results. Key lessons from our methods can contribute toward best practices for a UCD approach to develop smart, pervasive AT for dementia. Our findings in terms of usability, usefulness and user adoption can be used to improve future design endeavours.

### 5.1 UCD for developing smart, pervasive AT

We have employed a user-centred approach in the development and testing of a pervasive AT solution. Inclusive design guided the selection technology for a first simple prototype even before end-users were involved by relating specifications (e.g. screen size and buttons) to users’ capabilities. More importantly, we have demonstrated how involving end-users in various testing methods can generate valuable design insights.

The combination of methods used provided information on three different aspects of usability: perceived overall usability, actual usability for different support features and usability issues. These complemented each other, providing distinct and valuable information at increasing levels of detail. SUS scores gave an impression of overall usability from the end-user's perspective. Task completion rates – an indication of actual usability – highlighted which support features were more usable than others. Investigating specific usability issues added depth to these results by providing clues as to *why* these features were not usable, offering directions for future designs.

The importance of field testing ‘in the wild’ was highlighted by the compared SUS scores. These tended to be both lower and more similar following the field testing, suggesting a more realistic indication of users’ perceptions. The field testing also allowed deeper insight into usefulness, since it depends on users choosing to use the solution (unlike with prescribed tasks or trying to imagine whether they would use it). In summary, we recommend the following practices for user-centred development of pervasive AT:

- Including both controlled and real-world testing conditions.
- Combining subjective data (participants perceptions) with objective measures.
- Using multiple testing methods for varying levels of detail on key issues and their possible causes.

### 5.2 Usability, usefulness and user acceptance

Usability and usefulness were focal points of this research because of their known influence on adoption among elderly users. The user testing yielded interesting findings in each of these areas.

Regarding perceived overall usability, the participant without previous experience using a smartphone (P3) was particularly noteworthy. Despite extremely poor initial perceptions of usability (SUS score), this improved to the level of the other participants after a week's field testing. This promising result indicates that experience is not a prerequisite for smart technology to be perceived as usable.

For specific support features, our findings suggest that the *scheduling*, *communication* and *orientation* features were usable, whereas the *navigation* and *emergency help* features were clearly not usable at all. Digging deeper into the issues that inhibit usability raises the question of who should use this information. Here, we recognise a distinction between designers of the existing hardware or software, and designers of the pervasive AT concept. Issues such as device pairing or battery charging can inform the design of the pervasive technologies, such that they might be more inclusive in future. Issues such as using multiple apps for a single support feature or accidentally recording blank Google Keep notes can inform the customisation of these pervasive technologies to serve as AT for PwD. A key takeaway was that the smartwatch has poor usability as an input device, and should be used for notifications, orientation and possibly monitoring (gathering sensor data) only.

Regarding usefulness, again *scheduling* stands out as most appealing and *navigation* the least. This may be due to the PwD being in the early stages, during which getting lost may not be a concern. Usefulness was a draw factor for the two adopters, underscoring the need to meet users’ needs. Overall, our findings show some promise for user adoption of the pervasive AT solution, with usability and usefulness both being significant factors.

Two central aspects that emerged were *familiarity* and *personalisation*. Participants tend to prefer what they are familiar with – a *‘go with what you know’* attitude. This is evident in the tendency to choose the contact book over the calling shortcut to call their caregiver, confusion over swiping (which may not be as familiar as pressing buttons) and a preference for one's current scheduling solutions. Choosing hardware and software that are most aligned with what users are used to could therefore improve both usability and perceived usefulness, and ultimately adoption. Regarding personalisation, a standard set of support features was tested rather than a set tailored to each participant's preferences. By including only those solutions that the user is interested in, the solution may be less overwhelming and seem more relevant. The following recommendations summarise the complete findings:

- The smartphone should be used for input and the smartwatch for output (e.g. notifications, orientation and behaviour sensing).
- Scheduling (including notes, reminders and notifications) are most promising in terms of both usability and usefulness.
- Navigation and emergency support were not perceived as useful or usable.
- Familiarity is an important design consideration since users tend to have a *go with what you know* approach.
- The solution should be personalised to include only those support features that the user deems relevant for their individual needs.

### 5.3 Limitations and future work

The user testing methods were time consuming in terms of data collection and analysis, limiting the number of participants. PwD vary substantially regarding symptoms, lifestyle, background and technology literacy etc. – all of which may influence adoption – therefore, it is not possible to make broad generalisations about user adoption of the pervasive AT solution. Instead, our results provide a first impression, showing some promise for user adoption and pinpointing key issues for further study.

Extending the field testing over months would allow greater insight into use patterns than were possible for 1 week. Long-term testing could be used to explore whether use and acceptance improve over time with learning and habit or whether it drops as the novelty wears off or the user's condition worsens, and uncover issues such as whether users are able to routinely charge the devices. Another interesting avenue would be to tailor the pervasive AT to individual needs, which was excluded to allow comparison of the same solution across participants.

Regarding the pervasive AT solution, though highly promising, this cannot replace all other forms of AT (e.g. home automation equipment or animaloid robots). Furthermore, while a wrist-worn device maybe easier to remember than a smartphone, it is not fool-proof. Removing and forgetting the device is still a concern. The monitoring capabilities were not tested in this work. Behavioural measurement could support timely and targeted intervention, improving the quality of care; therefore, extensive research – including user testing methods – are required in this area.

Finally, implementation in clinical practice is emphasised as an important area of future research, since this is a significant challenge for wearable technology in healthcare [32]. Strategies for healthcare providers to introduce pervasive AT to PwD and integrate them into care practices are needed to accelerate and support adoption.

## 6 Conclusion

This work describes the development and testing of a pervasive AT solution created by combining off-the-shelf smart technology. These devices offer advantages over specialised AT regarding user adoption, motivating an evaluation of their usability, usefulness and user acceptance.

A smartphone, smartwatch and several apps were combined to offer six support features including: scheduling, communication, orientation, navigation, emergency help and monitoring. This was tested with five participants with mild dementia and their spouses through usability testing and field testing. SUS scores, task completion rates, interaction logs and user feedback were analysed to evaluate perceived and actual usability, and to pinpoint key usability issues. Logged usage and semi-structured interviews based on the UTAUT model were analysed to assess usefulness and to identify four main adoption profiles.

The results showed promise for user adoption overall, with scheduling as most successful and navigation least in terms of usability and usefulness. On the basis of our findings, we contribute with suggested UCD practices for developing pervasive AT such as combining test methods, including end-users’ perceptions and testing in a real-world context. A set of recommendations for future designs is also provided based on our results including: using the smartwatch as output only, personalising the solution to users’ individual needs, and making it as familiar to them as possible. There are still challenges ahead that call for further research on long-term adoption patterns, behavioural monitoring and clinical implementation. The ultimate vision is pervasive, person-centred care to support PwD and improve their quality of life.
